# The Salmon Oil OmeGo Reduces Viability of Colorectal Cancer Cells and Potentiates the Anti-Cancer Effect of 5-FU

**DOI:** 10.3390/md21120636

**Published:** 2023-12-12

**Authors:** Caroline H. H. Pettersen, Helle Samdal, Pål Sætrom, Arne Wibe, Erland Hermansen, Svanhild A. Schønberg

**Affiliations:** 1 Department of Clinical and Molecular Medicine, Faculty of Medicine and Health Sciences, Norwegian University of Science and Technology (NTNU), 7491 Trondheim, Norway; helle.samdal@ntnu.no (H.S.); pal.satrom@ntnu.no (P.S.); arne.wibe@ntnu.no (A.W.); svanhild.schonberg@ntnu.no (S.A.S.); 2 Department of Surgery, St. Olav’s University Hospital, 7006 Trondheim, Norway; 3 Hofseth BioCare, Kipervikgata 13, 6003 Ålesund, Norway; ehe@hofsethbiocare.no; 4 Department of Computer Science, Faculty of Information Technology and Electrical Engineering, Norwegian University of Science and Technology (NTNU), 7491 Trondheim, Norway; 5 Bioinformatics Core Facility—BioCore, Norwegian University of Science and Technology (NTNU), 7006 Trondheim, Norway; 6 K.G. Jebsen Center for Genetic Epidemiology, Norwegian University of Science and Technology (NTNU), 7006 Trondheim, Norway

**Keywords:** colorectal cancer, CRC, fish oil, omega-3 fatty acids, salmon oil, OmeGo

## Abstract

Colorectal cancer (CRC) is one of the most common cancer types worldwide. Chemotherapy is toxic to normal cells, and combinatory treatment with natural well-tolerated products is being explored. Some omega-3 polyunsaturated fatty acids (n-3 PUFAs) and marine fish oils have anti-cancer effects on CRC cells. The salmon oil OmeGo (Hofseth BioCare) contains a spectrum of fatty acids, including the n-3 PUFAs docosahexaenoic acid (DHA) and eicosahexaenoic acid (EPA). We explored a potential anti-cancer effect of OmeGo on the four CRC cell lines DLD-1, HCT-8, LS411N, and LS513, alone and in combination with the chemotherapeutic agent 5-Fluorouracil (5-FU). Screening indicated a time- and dose-dependent effect of OmeGo on the viability of the DLD-1 and LS513 CRC cell lines. Treatment with 5-FU and OmeGo (IC20–IC30) alone indicated a significant reduction in viability. A combinatory treatment with OmeGo and 5-FU resulted in a further reduction in viability in DLD-1 and LS513 cells. Treatment of CRC cells with DHA + EPA in a concentration corresponding to the content in OmeGo alone or combined with 5-FU significantly reduced viability of all four CRC cell lines tested. The lowest concentration of OmeGo reduced viability to a higher degree both alone and in combination with 5-FU compared to the corresponding concentrations of DHA + EPA in three of the cell lines. Results suggest that a combination of OmeGo and 5-FU could have a potential as an alternative anti-cancer therapy for patients with CRC.

## 1. Introduction

The outcome of colorectal cancer (CRC) has improved over the past decades; however, it is still the second and third most common cancer type worldwide among women and men, respectively. In Norway, CRC is the second most common cancer type for men and women together, with approximately 4500 new cases every year [1]. The incidence rate is high in Norway compared to other Nordic countries and has increased by 300% in the last 60 years [2]. In 2014, CRC was the costliest cancer in Norway, constituting a significant economic burden on Norwegian society [3].

For treatment of CRC, surgery is still the cornerstone of curative intent [4]. However, adjuvant cancer chemotherapy is commonly used after resection of advanced tumors [5]. One of the first-line chemotherapy drugs for CRC treatment is 5-fluorouracil (5-FU), which is commonly used alone or in combination with other anti-cancer drugs. Although 5-FU is considered one of the safest chemotherapy agents, chemotherapy may lead to development of drug resistance and toxicity towards normal cells [6]. This has led to an increased interest in exploring the potential anti-cancer effects of different natural dietary ingredients such as fish and fish oil in combination with chemotherapy to improve CRC treatment and patient quality of life. 

Marine fish oil is a good dietary source of the omega-3 (n-3) polyunsaturated fatty acids (PUFAs) docosahexaenoic acid (DHA) and eicosapentaenoic acid (EPA), which have been shown to have anti-cancer properties (reviewed in [7,8]). The human body has limited capacity to synthesize these PUFAs; hence, they are considered essential and must be acquired through the diet. The Norwegian authorities recommend a daily intake of at least 1–2 g n-3 PUFAs or two to three fatty fish meals weekly [9], while the European Food Safety Authority (EFSA) recommends an intake of ≥2 fish meals weekly or an intake of 250–500 mg DHA and EPA daily [10]. However, both the Norwegian and American intake of DHA and EPA is below the recommended levels, and intake of fatty fish or fish oils should be increased to improve health [11,12].

Some epidemiological studies suggest that the intake of n-3 PUFAs reduces the risk of developing CRC [13] and that intake of fish and n-3 PUFAs may have the potential to affect the outcome of CRC treatment [14]. Observational data indicate reduced mortality after CRC diagnosis, and longer disease-free survival, in patients with high intake of n-3 PUFAs [15,16]. Some interventional studies demonstrate beneficial anti-cancer effects of n-3 PUFAs in CRC patients [17,18] and EPA supplementation was shown to reduce crypt cell hyperproliferation and increase mucosal apoptosis in patients with colorectal adenomas [18,19]. EPA supplementation given pre-surgically to patients with CRC metastases improved overall and disease-free survival compared to placebo [20]. Also, intake of n-3 PUFA-containing perioperative nutrition may reduce postoperative complications, pro-inflammatory cytokine levels and hospital stay for CRC patients [21]. Animal studies indicate that n-3 PUFAs and fish oil may decrease the formation and growth of CRC tumors in vivo [7,22,23] and improve the efficacy of chemotherapeutic drugs like 5-FU [7,14,22], and that fish oil may increase cellular uptake of 5-FU in the colon of mice, thereby reversing multi-drug resistance and restore 5-FU-mediated chemosensitivity [24]. In vitro studies confirm the anti-cancer potential of n-3 PUFAs alone and in combination with chemotherapeutic drugs like 5-FU and suggest a range of different molecular pathways involved [7,14,22,25]. Commercially, n-3 PUFAs are available in different formulations: as free fatty acids (FAs), phospholipids, triglycerides, and conjugated to ethyl esters. The effect of different formulations alone and in combination with chemotherapeutic drugs on cancer cells may vary [14]. DHA and EPA enhance the anti-cancer effect of chemotherapies on human cancer cells both in their pure forms [25,26,27,28,29,30] and when delivered as part of liposomes [31]. Fish oils may also enhance the effect of cytostatic treatment on cancer cells [32,33,34,35]. 

In this study, we tested a potential anti-cancer effect of the salmon oil OmeGo on CRC cells alone and in combination with the chemotherapeutic drug 5-FU. We also compared the effect to corresponding concentrations of the n-3 PUFAs DHA and EPA. We selected two DHA-sensitive cell lines, DLD-1 and HCT-8, and two less sensitive cell lines, LS411N and LS513. The choice of cell lines was based on a previous publication from our group where we tested the DHA sensitivity and basal level of autophagy on 10 CRC cell lines that represented different clinically relevant subtypes [36]. OmeGo is a natural fish oil liberated from Atlantic salmon using a patented enzymatic process (Hofseth BioCare). OmeGo contains ~99% fat, of which less than 1% is free FAs (21 different identified) and about 1% lipopeptides, and meets the FDA standards for New Dietary Ingredients (NDI) status [37]. Taken as Cardio capsules, it contains about 140 mg n-3 PUFAs per gram of salmon oil [38]. It also contains the natural carotenoid astaxanthin, an antioxidant, which originates from algal production and gives the red color of the salmon oil [39]. OmeGo has in previous studies demonstrated an anti-eosinophilic effect and may have beneficial effects on eosinophil-driven diseases such as asthma and Chronic obstructive pulmonary disease (COPD) [40,41], as well as cardio-vascular events through reduction of serum concentrations of Oxidized low-density lipoprotein-2-glycoprotein I complex x (oxLDL-GP) [42].

## 2. Results

### 2.1. 5-FU and OmeGo Treatment Reduce Viability of CRC Cell Lines

**Figure 1:**
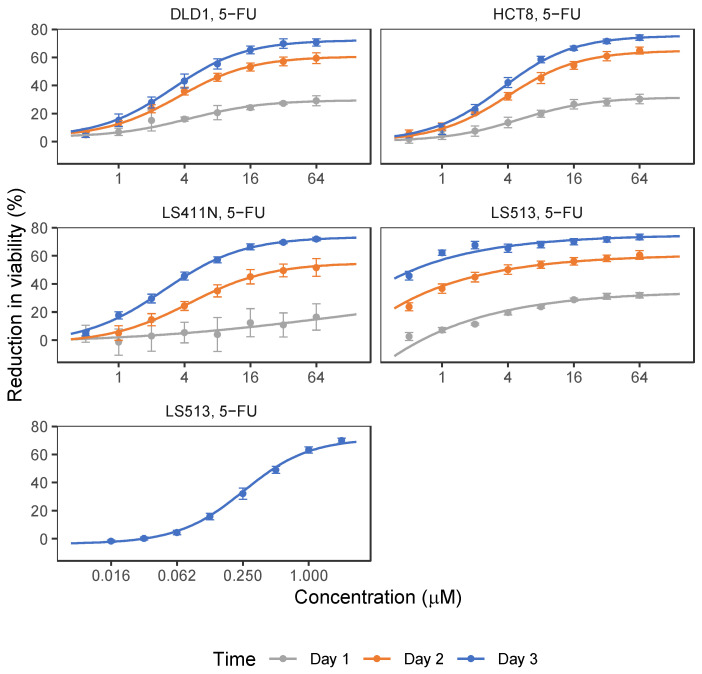
Effect of 5-Fluoruracil (5-FU) treatment on the DLD-1, LS411N, HCT-8, and LS513 cell lines. Points show average percent reduction of cell viability after treatment with indicated concentrations of 5-FU for 1–3 days. Error bars show standard deviation (SD). Lines show fitted dose–response curves (see Materials and Methods section). Number of biological replicates (*n*) for all 4 cell lines (0.5–64 µM 5-FU) = 4. For LS513 (0.015625–2 µM) *n* = 3.

Four CRC cell lines were selected for testing the potential anti-cancer effect of OmeGo alone and in combination with 5-FU using the Resazurin viability assay. 5-FU was tested in the range of 0.5–64 µM based on previously reported blood [5-FU] in cancer patients undergoing 5-FU treatment (2.54–17.4 µM) [43,44] and previously reported [5-FU] tested on CRC cells in vitro [27,34,45,46]. 5-FU treatment reduced the viability of all four cell lines in a time- and dose-dependent manner (*p* < 2.4 ×10^−56^, Wald tests) (1–3 days, Figure 1, Supplementary Table S1). The reduction in cell viability did not exceed 70% for the highest [5-FU] tested (3 days) for all cell lines. LS513 cells were highly sensitive to 5-FU treatment; hence, the [5-FU] used for screening was reduced as indicated in Table S1 and Figure 1 to find the linear area of the dose–response curve for this cell line.

**Figure 2:**
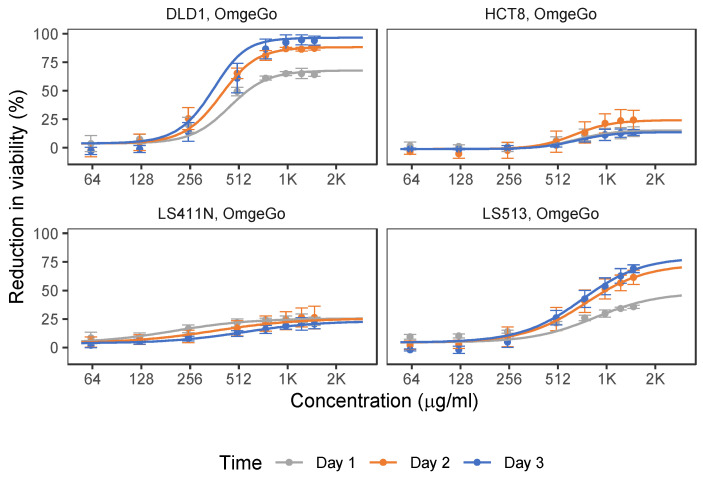
Effect of OmeGo treatment of the DLD-1, LS411N, HCT-8, and LS513 cell lines. Points show average percent reduction of cell viability after treatment with indicated concentrations of OmeGo for 1–3 days (DLD-1 and LS513 *n* = 4, LS411N and HCT-8 *n* = 5). Error bars show standard deviation (SD). Lines show fitted dose–response curves (see Materials and Methods section).

Screening of the anti-cancer effect of OmeGo was performed in the range of 62.5–1500 µg/mL based on estimated DHA and EPA content in OmeGo and previous reported doses of DHA, EPA, and fish oil tested on cancer cells [36,47,48,49]. OmeGo treatment reduced cell viability in a time- and dose-dependent manner with up to 90% and 70% for the DLD-1 and LS513 cell lines, respectively (Figure 2, Table S2; *p* < 4.9 × 10^−128^, Wald tests). For DLD-1 cells, the linear area of the sensitivity curve was between 125–750 µg/mL but flattened out above 1000 µg/mL OmeGo. For LS513 cells, the sensitivity was still increasing up to 1500 µg/mL. The HCT-8 and LS411N cell lines responded much less to OmeGo treatment, with a maximum reduced viability of ~24% and ~26% after 2 days, respectively (Figure 2, Table S2; *p* < 8.4 × 10^−51^, Wald tests).

The concentrations of 5-FU and OmeGo that gave 20%, 30% and 50% of the maximal measured effect on cell viability (IC20, IC30, and IC50) after 3 days were estimated for all cell lines (Table 1). The IC50 values for 5-FU treatment of DLD-1, HCT-8, and LS411N cells ranged from 4.6 µM to 5.9 µM (Table 1). The initially estimated 5-FU IC20–IC50 values for LS513 cells were below the concentrations tested due to high sensitivity, and the data did not fit the model well. However, the estimated IC values (IC20 = 0.005 ± 0.00, IC30 = 0.013 ± 0.00) guided the choice of concentrations for an additional screening of LS513 cells using lower concentrations of 5-FU (0.0156–2.0 µM, 3 days, Table 1, Figure 2), resulting in an IC50-value of 0.4 µM for this cell line.

### 2.2. OmeGo Treatment Potentiates the Anti-Cancer Effect of 5-FU in CRC Cell Lines

**Figure 3:**
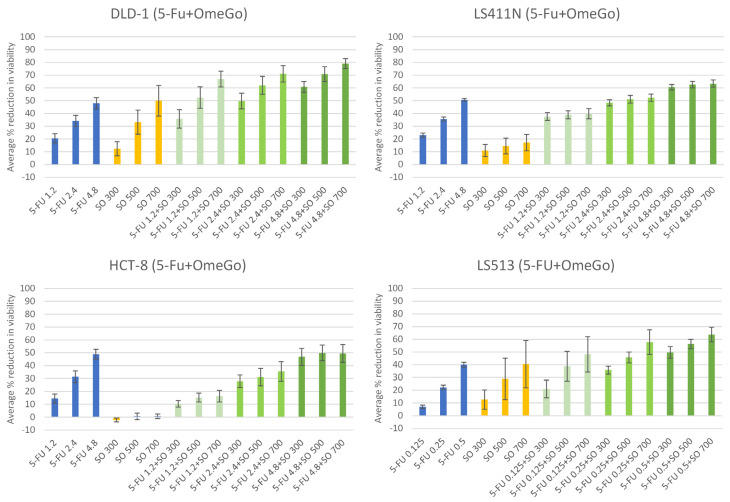
Effect of 5-FU (blue), OmeGo (SO = salmon oil, orange), and combinatory treatment with 5-FU + OmeGo (SO) (green) on viability of the DLD-1, LS411N, HCT-8, and LS513 cell lines. Results represent average percent reduction of cell viability (±SD) after treatment with indicated concentrations of 5-FU (µM) and OmeGo (SO, µg/mL) for 3 days (DLD-1, LS411N and HCT-8 *n* = 5, LS513 *n* = 4).

For combination experiments, 5-FU and OmeGo were used in concentrations covering the IC20–IC30 ranges for all cell lines (Figure 3 and Table 1). Statistical analyses indicated a significant dose-dependent effect of 5-FU treatment for all cell lines (Figure 3, Table 2). Each unit 5-FU (µM) was estimated to reduce cell viability, with 7.7–9.8% for DLD-1, HCT-8 and LS411N cells, while viability in the highly 5-FU sensitive LS513 cells was reduced by 88.5% per µM (Figure 3, Table 2 and Table S3). OmeGo treatment had a significant dose-dependent additive effect in DLD-1 and LS513 cells (Figure 3, Table 2), where each unit of OmeGo (100 µg/mL) was estimated to reduce viability by about 6%. Cotreatment with 5-FU and OmeGo had a small significant antagonistic interaction in DLD-1 and LS513 cells; however, it further reduced the cell viability for DLD-1 (*p*-value = 0.039) and LS513 cells (*p* = 1.0 × 10^−3^), respectively (Table 2, Figure S1). Based on data presented in Figure 3, combinatory treatment with the lowest [5-FU] combined with different [OmeGo] gave an increased effect of 15–46%, while the highest [5-FU] combined with different [OmeGo] increased the effect by 13–31%, compared to the respective [5-FU] alone for DLD-1 cells (Table S3). For LS513 cells, the lowest [5-FU] combined with different [OmeGo] gave an increased effect of 14–41%, while the highest [5-FU] combined with different [OmeGo] increased the effect by 10–24% (Table S3). Hence, the largest chemo-sensitizing effect of OmeGo was seen at low [5-FU].

### 2.3. The n-3 PUFAs DHA + EPA Potentiate the Anti-Cancer Effect of 5-FU in CRC Cells

**Figure 4:**
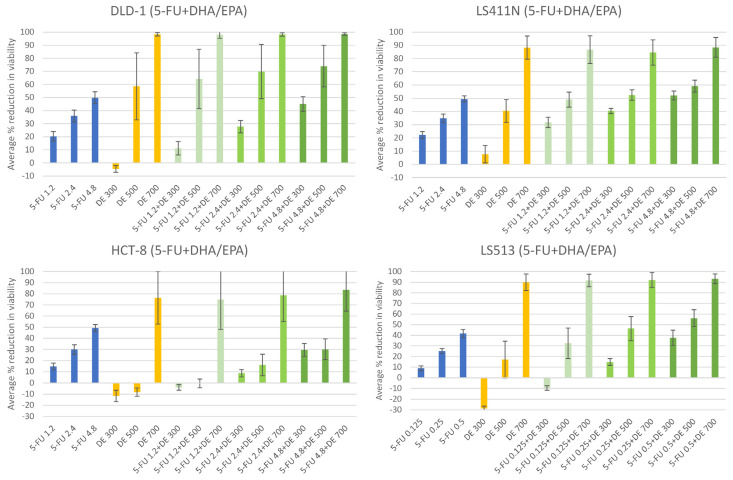
Effect of 5-FU (blue), docosahexaenoil acid (DHA) + eicosapentaenoic acid (EPA) (DE, orange), and combinatory treatment with 5-FU+DE (green) on viability of the DLD-1, LS411N, HCT-8, and LS513 cell lines. Results represent average percent reduction of cell viability (±SD) after treatment with indicated concentrations of 5-FU (µM) and DE (dose correlating with µM/mL OmeGo) for 3 days (DLD-1 and LS411N *n* = 6, HCT-8 and LS513 *n* = 5).

The content of DHA and EPA in 1 mg OmeGo was estimated to be 124 µM and 95.2 µM, respectively (based on information in the OmeGo certificate of analysis). Based on this, concentrations of DHA and EPA corresponding with doses of 300, 500, and 700 µg/mL OmeGo were used for combinatory treatment with 5-FU (Figure 4, Table S5). The lowest [DHA + EPA] somewhat enhanced cell viability (Figure 4, Table S6), except for LS411N cells, and hence had a less negative effect on viability compared to the corresponding concentration of OmeGo (Figure 2 and Figure 3; *p* = 9.0 × 10^−7^, Student’s *t*-test). The combination of the lowest [5-FU] and [DHA + EPA] reduced cell viability to a lesser extent compared to 5-FU alone for the DLD-1, HCT-8 and LS513 cells (*p* < 2.1 × 10^−6^, Student’s *t*-test) and did not reach the level of the combinatory effect of 5-FU+OmeGo at corresponding concentrations (Figure 3 and Figure 4; *p* = 5.0 × 10^−10^, Student’s *t*-test). The highest [DHA + EPA] (~153 µM n-3 PUFAs, Table S5) seemed to be toxic to some cell lines (Figure 4, Table S6). 

Statistical analyses indicated that DHA + EPA treatment reduced viability for all cell lines within concentrations present in the OmeGo IC20–IC30 range. Viability was reduced by 9–14% per 100 µg/mL OmeGo-correlated concentration of DHA + EPA. The 5-FU also reduced viability by 11–14% for DLD-1, HCT-8, and LS411N cells, and 150% for the highly sensitive LS513 cells, per µM 5-FU treatment (Table 3). The combinatory treatment with DHA + EPA and 5-FU had a small significant antagonistic interaction in all cell lines (Table 3, Figure S2). The effect of DHA + EPA and the combinatory treatment estimated by the linear model diverged more from the observed effects (Figure 4, Tables S6 and S7) compared to the OmeGo results, probably reflecting the less optimized dosage of n-3 PUFAs compared to OmeGo.

## 3. Discussion

There is an increased interest in testing natural dietary compounds for potential anti-cancer effects both alone and in combination with already established cancer therapies. In this study, we found the viability of four tested CRC cell lines to be reduced by treatment with the chemotherapeutic agent 5-FU in a time- and concentration-dependent manner, which is in accordance with previous findings [50,51]. The LS513 cells were highly sensitive to 5-FU treatment compared to the other cell lines tested. This is consistent with the results by Bracht et al., who showed that LS513 cells were more sensitive to 5-FU compared to the DLD-1 and LS411N cells [52]. Testing a potential anti-cancer effect of the salmon oil OmeGo (HBC) showed that OmeGo reduced viability of two of the four tested CRC cell lines in a time- and dose-dependent manner. A combinatory treatment with 5-FU and OmeGo resulted in a further reduction in cell viability compared to 5-FU alone and hence chemosensitization of these CRC cell lines to 5-FU treatment. This indicates that OmeGo may be effective as an adjuvant or chemosensitizer together with chemotherapeutic agents to enhance the effectiveness of conventional CRC therapies.

The potential of fish oils to enhance the effect of chemotherapeutic agents like 5-FU has also been found by others. Granci et al. showed that a fish oil emulsion enhanced the cytotoxic and apoptosis-inducing effect of 5-FU in one of two CRC cell lines [33], while Jordan et al. found a fish oil-based lipid emulsion to enhance 5-FU-induced growth inhibition of CRC cells [34]. Rani and colleagues found that fish oil chemosensitized CRC cells to 5-FU treatment in animal models [24,35,53]. Studies also show the potential of the marine n-3 PUFAs DHA and EPA to enhance the anti-tumor effect and reduce cytotoxic effects of chemotherapeutics like 5-FU both in vitro in CRC cell lines and in animal models as reviewed by Hull et al. [14]. 

To compare the effect of OmeGo to the effect of the free omega-3 PUFAs DHA and EPA, the cells were treated with DHA and EPA concentrations corresponding with the estimated DHA and EPA levels in OmeGo. In contrast to OmeGo, the lowest DHA + EPA concentration tended to slightly stimulate cell viability in some cell lines. This was also seen for the effect of the combinatory treatment with the lowest concentration of 5-FU and DHA + EPA for DLD-1, HCT-8, and LS513 cells. The combinatory treatment reduced cell viability compared to treatment with DHA + EPA alone, but to a lesser extent than 5-FU treatment alone. When a linear model was fitted to the data, the free n-3 PUFAs were estimated to have a higher effect per unit compared to OmeGo (100 µg/mL). However, this probably reflects the extensive reduction in viability of the highest concentration of the free n-3 PUFAs, which seemed toxic to the cells. The estimated effects of DHA + EPA alone and in combination with 5-FU diverted from the observed effect, especially for the lower concentrations, indicating an additive but not linear effect for the n-3 PUFAs. Somehow, it seemed to be a threshold value for treatment with the n-3 PUFAs, with an enhanced reduction of cell viability when crossing the threshold. This is also reflected by the high standard deviations for some of the cell lines after DHA + EPA treatment. The highest dose of DHA + EPA (~150 µM) is high compared to the basal plasma total concentrations of DHA (~80 µM) and EPA (~20 µM), although such plasma concentration may be achieved by DHA/EPA and/or fish oil supplements [54]. However, as stated by Serini et al. based on in vivo results and the fact that cancer cells have different sensitivity to the cytotoxic effects of n-3 PUFAs, they never use n-3 PUFA concentrations over 30–50 µM in their experiments [55]. In contrast to OmeGo, the lower doses of n-3 PUFAs, which would correspond to typical physiological doses, did not enhance the effect of 5-FU in terms of reduced cell viability. The highest concentration of OmeGo showed a more balanced effect on cell viability and hence may be used as an adjuvant to cancer cell therapies in concentrations that are not physiologically relevant for DHA/EPA alone.

Why some cancer cells are sensitive towards n-3 PUFAs, while others are not, is still unknown. We are currently addressing this in an ongoing study where we investigate genetic differences that may affect n-3 PUFA sensitivity in cancer cells. In a previous publication from our group, we found that DHA sensitivity correlated with a specific gene expression profile, the basal levels of autophagy, and MAP1LC3B-II protein in 10 different CRC cell lines [36].The tested CRC cell lines responded very differently towards DHA treatment; the DLD-1 and HCT-8 cells were about 50% and 30% growth-inhibited by DHA (70 µM) treatment for 48 h, respectively, compared to no (or a slightly positive) effect on growth of LS411N and LS513 cells under the same conditions (assessed by cell counting) [36]. The results presented here indicate less effect of the combination of corresponding [DHA + EPA] on DLD-1 and HCT-8 cells compared to previous results with DHA treatment alone. However, the combination of DHA + EPA might have a different effect on the cells compared to DHA alone, and we previously showed that EPA has a somewhat lower effect on CRC cell lines compared to DHA [56]. The type of growth media used was the same as previously. However, we changed the type of fetal bovine serum (FBS) used, which might influence the results on n-3 PUFA sensitivity due to unknown factors such as level and type of growth factors and selenium. Selenium levels are known to vary between serum types and batches and may result in different responses of cancer cells to stress-causing agents [57]. Also, the Resazurin assay may not be directly compared with cell-counting results, as the capacity to reduce resazurin to resorufin is affected by the cells’ mitochondrial enzymes and metabolic capacity [58]. 

Treatment with different chemotherapeutic agents like 5-FU [59] and n-3 PUFAs is known to induce oxidative stress in human CRC cells [36,60]. The highest concentration of DHA + EPA prompted a very high reduction in cell viability for all the tested CRC cell lines, which might indicate induction of a high level of oxidative stress or cytotoxicity. The corresponding dose of OmeGo (700 µg/mL) had a lesser effect on cell viability. However, OmeGo contains the natural antioxidant and liposoluble carotenoid astaxanthin, which might reduce the oxidation of the n-3 PUFAs in OmeGo and/or the possibility of inducing oxidative stress in the treated cells. Astaxanthin has both antioxidant and anti-inflammatory activity (reviewed in [61]) and may also suppress CRC metastasis [62].

We are planning a follow-up study on molecular pathways affected by OmeGo treatment in CRC cells. Pre-clinical testing of potential new treatment regimens for CRC is highly needed, and we plan to continue the exploration of the anti-cancer potential of OmeGo in pre-clinical xenograft studies in mice. Only a few clinical trials have explored the anti-cancer effect of n-3 PUFAs and marine oils on CRC. However, some studies have reported an association between increased intake of marine n-3 PUFAs after CRC diagnosis and lower CRC-specific mortality [15] and longer disease-free survival for CRC patients with a high intake of dark-meat fish after diagnosis [16]. This will be interesting to study in a randomized intervention trial for patients with CRC given OmeGo in addition to conventional CRC treatment.

## 4. Materials and Methods

### 4.1. Cell Lines, Culture Conditions, and Chemicals

The CRC cell lines DLD-1, LS411N, HCT-8 and LS513 from American Type Tissue Collection (ATTC, Rockville, MD, USA) were grown in RPMI media (Gibco A1049101, Life Technologies, Carlsbad, CA, USA) in a humidified atmosphere at 5% CO_2_ and 37 °C. To the RPMI media was added fetal bovine serum (10%, Sigma #F7524, batch 0001660391, Sigma-Aldrich, Saint-Louis, MO, USA) and gentamicin (Gibco #1570049, Life Technologies). Cell lines were used up to passage ~20. Stock solution of OmeGo (Hofseth BioCare, Ålesund, Norway) was prepared in ethanol (1:8, 0.116 g/mL) and 5-FU (#548357, 50 mg/mL, Accord Healthcare AB, Harrow, UK) in phosphate-buffered saline (PBS, 0,0192 M); hence, both EtOH and PBS were used as vehicles. The OmeGo stock was stored at −20 °C, while the 5-FU stock was freshly prepared for each experiment. Stocks of DHA (Sigma-Aldrich, #D2534) and EPA (Sigma-Aldrich, #E2011) diluted in ethanol were stored at −20 °C. The dilution ratio of OmeGo in ethanol was optimized to assure a low effect of the vehicle, and the ethanol concentration did not exceed 0.75% volume/volume during treatments. For OmeGo, the same batch was used in all experiments.

### 4.2. Cell Treatment and Resazurin Viability Assay

Cells were seeded in 96-well trays (1500 cells/well) and incubated for 24 h before treatment with OmeGo, 5-FU, DHA, and EPA diluted in growth media in the concentrations given in the Results section. Cell viability was assessed using the Resazurin (7-Hydroxy-3H-phenoxazin-3-one 10-oxide) assay after 0, 24, 48 and 72 h (0–3 days) treatment. Media was removed and wells were washed once with PBS before adding resazurin (0.03 g/L) diluted in growth media. The resazurin stock was prepared in sterile 1 × PBS (0.15 g/L) and stored at −20 °C. Resazurin is a blue dye that is highly fluorescent when reduced to pink resorufin, which is proportional to aerobic respiration and the number of viable cells. The plates were incubated at 37 °C for 4 h before measuring fluorescence with a 544 nm excitation wavelength and a 590 nm emission wavelength using the FLUOstar Omega plate reader (BMG Labtech, Ortenberg, Germany).

### 4.3. Data Analysis

The average blank fluorescence signal was subtracted from the average fluorescence signal for each treatment before calculation of percent reduction of cell viability as percentage of signal compared to control. Dose–response curves were fitted to the cell viability data, and IC20, IC30, and IC50 values were estimated based on the resulting curves by using the functions drm and ED, respectively, from the R-package drc (version 4.1.3) [63]. We used the following log-logistic model to fit dose–response curves for each cell line and each treatment:

(1) $$c+\frac{d-c}{1+e^{b(\ln x-\ln e)}},$$

where parameters *b*, *c, d*, and *e* are the slope, lower limit, upper limit, and IC50, respectively. Two models were fitted to the data: a full model with a common upper limit and slope, lower limit, and IC50 dependent on treatment time; and a simple model with common slope, upper limit, and IC50 and lower limit dependent on treatment time. The final choice between the full and simple models was based on which model best fitted the data. All models used Tukey’s biweight function for robust fitting. The simple model was used to test for time- and dose-dependent effects. Specifically, the function linear Hypothesis from the R-package *car* was used to do a *F* statistics-based Wald test of the null hypotheses that the upper limit is equal to the lower limit for the 24 h treatment, that the lower limit for the 24 h treatment is equal to the lower limit for the 48 h treatment, and that the lower limit for the 48 h treatment is equal to the lower limit for the 72 h treatment. 

For estimation of the treatment effect of 5-FU, OmeGo, DHA + EPA and combination treatments, the lmList function from the R-package nlme was used to fit the data from each cell line and treatment to the linear model:

% reduced cell viability = β_5-FU_ × 5-FU (µM) + β_SO_ × SO (100 µg/mL) + β_5-FU_ × _SO_ × 5-FUxSO + ε (2)

where 5-FU and SO are the concentrations of 5-FU and OmeGo or DHA + EPA used in the experiment, respectively. The fitted models were used to create isobolograms and estimate combination effects for selected effective doses. Combination effects were computed by using the Chou–Talalay combination index [64]. For comparing treatment effects of specific levels of OmeGo, DHA + EPA, and 5-FU, the lme function from the R-package nlme was used to fit a hierarchical linear model with treatment as fixed effect and cell-line as random effect. All statistical analyses were conducted in R (version 4.1.3).

## 5. Conclusions

OmeGo significantly reduced viability and potentiated the anti-cancer effect of 5-FU for the DLD-1 and LS513 CRC cell lines. Low doses of OmeGo had a higher negative effect on viability of CRC cells both alone and in combination with 5-FU compared to the corresponding lowest doses tested for DHA and EPA. Results suggest that treatment with a combination of OmeGo and 5-FU could be an alternative treatment strategy for patients with CRC. This will be further tested in pre-clinical and clinical studies. 

## Figures and Tables

**Table 1:** Estimated values for IC20, IC30, and IC50 ± standard error (SE) for CRC cell lines treated with 5-FU and OmeGo for 3 days.

Cell Line	Days	5-FU (µM)						OmeGo (µg/mL)					
		IC20	±SE	IC30	±SE	IC50	±SE	IC20	±SE	IC30	±SE	IC50	±SE
DLD-1	3	1.68	0.01	2.56	0.01	4.99	0.01	353.10	1.40	410.22	1.34	519.28	1.03
HCT-8	3	2.02	0.001	3.06	0.01	5.90	0.01	464.66	1.66	537.80	1.59	676.73	3.11
LS411N *	3	1.38	0.01	2.20	0.01	4.56	0.02	278.06	2.39	370.96	2.76	583.60	5.19
LS513	3	0.15	0.00	0.216	0.00	0.40	0.00	534.85	3.75	680.84	5.97	994.97	12.34

* For estimation of inhibitory concentration (IC20–IC50) values, the “robust” model was used for the LS411N cell line, while the “robust simple” model was used for the DLD-1, HCT-8, and LS513 cell lines, based on which model best fitted the data.

**Table 2:** Estimated percent (%) reduced viability (±SD) per unit treatment with 5-FU (µM), OmeGo (100 µg/mL) and combination of 5-FU + OmeGo of CRC cell lines (3 days). *p*-value < 0.05 was considered statistically significant.

Cell Line	Days	ε Intercept	5-FU			OmeGo			5-FU × OmeGo		
			Effect %	±SD	*p*-Value	Effect %	±SD	*p*-Value	Effect %	±SD	*p*-Value
DLD-1	3	8.55	8.94	0.77	1.17 × 10^−26^	6.17	0.52	9.94 × 10^−28^	−0.36	0.17	3.85 × 10^−2^
HCT-8	3	2.82	9.82	0.79	3.80 × 10^−29^	−0.02	0.53	9.67 × 10^−1^	0.07	0.18	6.88 × 10^−1^
LS411N	3	15.30	7.70	0.77	6.14 × 10^−21^	1.23	0.52	1.90 × 10^−2^	0.30	0.17	8.09 × 10^−2^
LS513	3	−2.17	88.46	8.14	9.55 × 10^−24^	6.29	0.57	3.64 × 10^−24^	−6.10	1.84	1.00 × 10^−3^

**Table 3:** Estimated % reduced viability (±SD) per unit treatment with 5-FU (µM), DHA + EPA (100 µg/mL OmeGo) and combination of 5-FU+ and DHA + EPA on CRC cell lines (3 days). *p*-value < 0.05 was considered statistically significant.

Cell Line	Days	ε Intercept	5-FU			DHA + EPA			5-FU × DHA + EPA		
			Effect %	±SD	*p*-Value	Effect %	±SD	*p*-Value	Effect %	±SD	*p*-Value
DLD-1	3	−10.86	12.45	2.07	4.32 × 10^−9^	13.79	1.36	2.15 × 10^−21^	−1.68	0.46	3.00 × 10^−4^
HCT-8	3	−20.26	13.76	2.19	1.00 × 10^−9^	9.03	1.46	1.65 × 10^−9^	−1.53	0.49	1.80 × 10^−3^
LS411N	3	−0.99	10.74	2.07	3.49 × 10^−7^	10.60	1.36	7.47 × 10^−14^	−1.40	0.46	2.20 × 10^−3^
LS513	3	−33.60	150.76	21.93	2.62 × 10^−11^	14.05	1.50	7.72 × 10^−19^	−17.31	4.81	4.00 × 10^−4^

## Data Availability

All relevant data is given in the manuscript and Supplementary Materials.

## Supplementary Materials

The following supporting information can be downloaded at: https://www.mdpi.com/article/10.3390/md21120636/s1, Table S1: Effect of 5-FU on CRC cell lines (average % reduction in cell viability (±SD)); Table S2: Effect of OmeGo on CRC cell lines (average % reduction in cell viability (±SD)); Table S3: Effect of combinatory treatment with OmeGo and 5-FU (3 days) on CRC cell lines (average % reduction in cell viability (±SD)); Table S4: Estimated effect of combinatory treatments of 5-FU and OmeGo in indicated concentrations; Table S5: Concentration of DHA and EPA (µM) corresponding to OmeGo doses (µg/mL) used for combinatory treatment with 5-FU; Table S6: Effect of combinatory treatment with DHA + EPA and 5-FU (3 days) on CRC cell lines (average % reduction in cell viability (±SD)); Table S7: Estimated effect of combinatory treatments of 5-FU and DHA + EPA in indicated concentrations (for DHA + EPA, the OmeGo correlated concentrations were used); Figure S1: Combination results from 5-FU and OmeGo treatment of DLD-1 and LS513 cells. For each cell line, the corresponding drug combination linear model was used to create OmeGo and 5-FU combination values for selected effective doses. Values are computed Chou–Talalay combination indices for selected drug combination values; Figure S2: Combination results from 5-FU and DHA + EPA treatment of DLD-1, LS411N, HCT-8, and LS513 cells. See Figure S1 for details.
