# Supplementary material for: The Salmon Oil OmeGo Reduces Viability of Colorectal Cancer Cells and Potentiates the Anti-Cancer Effect of 5-FU
Source: Mar Drugs. 2023 Dec 12;21(12):636. doi: 10.3390/md21120636 (PMC10744414; doi:10.3390/md21120636)
Supplement: Supplementary file 1 [file marinedrugs-21-00636-s001.zip › marinedrugs-2684828-supplementary.pdf]

Supplementary tables

Table S1. Effect of 5-FU on CRC cell lines (average % reduction in cell viability (±SD)). These are the data plotted in manuscript Figure 1.

Cell line	Days	0.5 µM	1 µM	2 µM	4 µM	8 µM	16 µM	32 µM	64 µM
**DLD-1**	1	6.24 (±2.74)	6.96 (±2.28)	15.14 (±6.42)	16.08 (±1.29)	20.62 (±4.40)	24.20 (±1.35)	27.32 (±0.78)	29.14 (±3.11)
	2	4.87 (±1.62)	13.02 (±1.70)	23.46 (±2.42)	35.96 (±2.42)	45.85 (±2.45)	53.13 (±2.43)	57.06 (±2.68)	59.39 (±3.30)
	3	5.27 (±1.91)	15.21 (±3.83)	27.94 (±3.44)	43.21 (±4.26)	55.38 (±3.09)	65.28 (±2.39)	69.82 (±2.96)	70.69 (±2.22)
**LS411N**	1	4.59 (±5.17)	-1.33 (±8.06)	3.01 (±9.38)	5.44 (±6.36)	4.08 (±10.50)	12.43 (±8.69)	10.91 (±7.40)	16.52 (±8.12)
	2	2.52 (±0.35)	5.07 (±4.48)	14.61 (±3.72)	24.39 (±2.80)	35.09 (±3.69)	45.17 (±4.48)	49.57 (±3.87)	51.71 (±5.49)
	3	4.84 (±1.57)	17.70 (±2.22)	29.65 (±2.73)	45.61 (±2.49)	57.20 (±1.79)	66.52 (±1.85)	69.65 (±0.76)	71.96 (±1.08)
**HCT-8**	1	1.83 (±2.42)	4.68 (±2.62)	7.54 (±3.14)	13.59 (±3.22)	19.91 (±2.16)	26.60 (±2.90)	28.11 (±2.36)	30.32 (±2.84)
	2	5.77 (±2.17)	10.67 (±2.18)	20.36 (±0.67)	32.30 (±2.29)	45.24 (±3.37)	54.31 (±2.42)	60.87 (±2.87)	64.85 (±2.21)
	3	3.13 (±1.05)	9.02 (±3.31)	22.89 (±3.02)	42.05 (±3.14)	58.45 (±1.93)	66.55 (±1.29)	71.44 (±1.37)	74.11 (±1.62)
**LS513**	1	2.63 (±2.48)	7.22 (±1.55)	11.46 (±0.67)	19.68 (±1.42)	23.76 (±0.80)	28.82 (±1.00)	31.25 (±1.83)	32.00 (±1.63)
	2	23.80 (±2.46)	36.75 (±2.86)	44.93 (±2.93)	50.19 (±2.89)	53.68 (±2.22)	56.15 (±2.32)	58.28 (±1.99)	60.61 (±2.64)
	3	45.84 (±2.66)	62.25 (±1.56)	67.65 (±2.38)	65.49 (±2.50)	68.06 (±2.02)	70.08 (±1.91)	71.68 (±1.62)	73.37 (±1.77)
		**0.0156 µM**	**0.0313 µM**	**0.0625 µM**	**0.125 µM**	**0.25 µM**	**0.5 µM**	**1 µM**	**2 µM**
**LS513**	3	-1.70 (±0.69)	0.21 (±0.89)	4.43 (±1.27)	15.81 (±1.92)	32.13 (±3.31)	49.05 (±2.02)	63.28 (±1.85)	69.85 (±1.45)

Table S2. Effect of OmeGo on CRC cell lines (average % reduction in cell viability (±SD)). These are the data plotted in manuscript Figure 2.

Cell line	Days	62,5 µM	125 µM	250 µM	500 µM	750 µM	1000 µM	1250 µM	1500 µM
**DLD-1**	1	3.66 (±6.06)	7.49 (±3.59)	20.10 (±5.73)	49.34 (±3.19)	61.05 (±1.52)	65.02 (±1.50)	65.06 (±3.89)	64.16 (±1.41)
	2	-2.00 (±5.20)	4.70 (±6.33)	25.59 (±8.32)	65.31 (±4.01)	80.96 (±3.72)	86.81 (±0.97)	86.38 (±1.41)	87.23 (±1.44)
	3	-2.75 (±2.70)	-1.17 (±2.57)	13.85 (±7.05)	61.05 (±11.15)	86.63 (±7.59)	92.34 (±5.72)	94.55 (±3.79)	93.88 (±3.35)
**LS411N**	1	8.37 (±4.52)	8.64 (±3.67)	16.34 (±2.96)	21.50 (±3.10)	24.06 (±1.45)	24.91 (±2.36)	25.60 (±3.38)	22.87 (±3.19)
	2	4.82 (±3.99)	6.85 (±3.79)	9.59 (±4.96)	16.82 (±5.91)	20.89 (±5.90)	23.44 (±7.14)	26.25 (±7.38)	26.18 (±8.95)
	3	1.88 (±1.96)	5.09 (±1.96)	7.74 (±1.91)	12.11 (±2.23)	15.90 (±3.15)	19.01 (±2.71)	20.71 (±4.87)	20.47 (±3.48)

**HCT-8**	1	1.67 (±3.03)	1.15 (±1.23)	0.62 (±3.56)	6.12 (±3.15)	11.21 (±4.55)	11.78 (±4.74)	12.86 (±4.40)	14.02 (±3.83)
	2	-3.39 (±2.02)	-5.21 (±3.59)	-2.28 (±6.18)	5.23 (±8.26)	13.52 (±8.35)	21.35 (±7.55)	23.67 (±8.69)	24.34 (±7.66)
	3	-1.02 (±0.67)	-1.25 (±0.54)	0.08 (±1.51)	2.32 (±1.44)	6.58 (±1.41)	11.18 (±4.41)	12.73 (±3.27)	13.42 (±2.22)
**LS513**	1	9.33 (±1.74)	10.10 (±1.56)	13.86 (±1.18)	21.41 (±1.09)	25.82 (±2.02)	29.79 (±2.68)	34.13 (±1.02)	35.67 (±1.25)
	2	1.95 (±3.20)	2.72 (±3.21)	9.32 (±7.53)	24.96 (±8.82)	40.58 (±8.53)	51.32 (±7.54)	56.55 (±5.97)	61.44 (±5.41)
	3	-2.17 (±0.72)	-2.04 (±2.72)	4.79 (±4.04)	25.99 (±5.55)	42.60 (±6.44)	53.55 (±6.42)	62.66 (±5.56)	68.97 (±2.95)

Table S3. Effect of combinatory treatment with OmeGo and 5-FU (3 days) on CRC cell lines (average % reduction in cell viability (±SD)). Numbers are based on raw data - blanc. These are the data plotted in manuscript Figure 3.

Cell line	Treatment	5-FU only	OmeGo 300	OmeGo 500	OmeGo 700
**DLD-1**	OmeGo only		12.40 (±5.59)	33.22 (±9.39)	49.97 (±12.03)
	5-FU 1.2 μM	20.52 (±3.56)	35.77 (±7.24)	52.48 (±8.55)	66.95 (±6.21)
	5-FU 2.4 μM	34.26 (±4.20)	49.82 (±6.05)	62.03 (±6.96)	71.02 (±6.36)
	5-FU 4.8 μM	48.02 (±4.40)	60.84 (±4.15)	70.80 (±5.82)	79.12 (±3.86)
**LS411N**	OmeGo only		11.02 (±4.84)	14.55 (±6.19)	17.25 (±6.34)
	5-FU 1.2 μM	23.07 (±1.59)	37.56 (±2.99)	39.01 (±3.07)	39.90 (±3.92)
	5-FU 2.4 μM	35.61 (±1.55)	48.42 (±2.46)	51.13 (±3.09)	52.40 (±2.83)
	5-FU 4.8 μM	50.72 (±1.20)	60.63 (±2.20)	62.67 (±2.59)	63.36 (±2.78)
**HCT-8**	OmeGo only		-2.25 (±1.53)	0.64 (±2.62)	0.69 (±1.79)
	5-FU 1.2 μM	14.52 (±3.52)	10.42 (±2.60)	15.30 (±3.47)	16.43 (±4.48)
	5-FU 2.4 μM	31.34 (±4.44)	27.89 (±4.75)	31.16 (±6.73)	35.54 (±7.51)
	5-FU 4.8 μM	48.87 (±3.77)	46.78 (±6.55)	49.84 (±6.09)	49.45 (±6.90)
**LS513**	OmeGo only		12.66 (±7.60)	28.88 (±16.29)	40.47 (±18.63)
	5-FU 0.125 μM	7.09 (±1.29)	21.01 (±6.95)	38.82 (±11.78)	48.10 (±13.91)
	5-FU 0.250 μM	22.24 (±1.74)	35.77 (±3.06)	45.73 (±4.30)	57.87 (±9.62)
	5-FU 0.500 μM	40.12 (±1.86)	49.65 (±4.49)	56.32 (±3.66)	63.75 (±5.76)

Table S4. Estimated effect of combinatory treatments of 5-FU and OmeGo in indicated concentrations. Values estimated by fitting the data to a linear model.

Cell line	Treatment	OmeGo 300	OmeGo 500	OmeGo 700
**DLD-1**	5-FU 1.2 μM	36.49	47.97	59.44
	5-FU 2.4 μM	45.92	56.54	67.15
	5-FU 4.8 μM	64.79	73.67	82.56
**LS411N**	5-FU 1.2 μM	29.31	32.49	35.67
	5-FU 2.4 μM	39.63	43.53	47.43
	5-FU 4.8 μM	60.27	65.61	70.95
**HCT-8**	5-FU 1.2 μM	14.80	14.92	15.05
	5-FU 2.4 μM	26.83	27.13	27.42
	5-FU 4.8 μM	50.90	51.54	52.17
**LS513**	5-FU 0.125 μM	25.47	36.53	47.58
	5-FU 0.250 μM	34.24	43.77	53.30
	5-FU 0.500 μM	51.78	58.26	64.74

Table S5. Concentration of DHA and EPA (μM) corresponding to OmeGo doses (μg/ml) used for combinatory treatment with 5-FU.

	OmeGo μg/ml		
**Fatty acid**	**300**	**500**	**700**
**EPA (μM)**	28.6	47.6	66.6
**DHA (μM)**	37.2	62.0	86.8
**Sum n-3 (μM)**	65.8	109.6	153.4

Table S6. Effect of combinatory treatment with DHA+EPA and 5-FU (3 days) on CRC cell lines (average % reduction in cell viability (±SD)). Numbers are based on raw data - blanc. These are the data plotted in manuscript Figure 4.

Cell line	Treatment	5-FU only	DHA+EPA 300	DHA+EPA 500	DHA+EPA 700
DLD-1	DHA+EPA only		-4.60 (±2.53)	58.59 (±25.61)	98.38 (±1.28)
	5-FU 1.2 μM	20.39 (±3.63)	11.17 (±5.17)	64.27 (±22.67)	97.82 (±2.49)
	5-FU 2.4 μM	35.90 (±4.59)	27.75 (±4.74)	69.89 (±20.69)	98.20 (±1.33)
	5-FU 4.8 μM	49.89 (±4.52)	45.04 (±5.64)	73.96 (±15.89)	98.56 (±0.98)
LS411N	DHA+EPA only		7.66 (±6.59)	40.35 (±8.70)	88.32 (±8.85)
	5-FU 1.2 μM	22.15 (±2.62)	31.71 (±3.95)	48.92 (±5.75)	86.73 (±10.49)
	5-FU 2.4 μM	34.82 (±3.24)	40.35 (±1.89)	52.38 (±3.94)	84.60 (±9.59)
	5-FU 4.8 μM	49.44 (±2.26)	52.11 (±3.24)	59.12 (±4.54)	88.47 (±7.53)
HCT-8	DHA+EPA only		-11.62 (±5.22)	-8.26 (±3.74)	76.41 (±23.71)
	5-FU 1.2 μM	14.72 (±3.03)	-4.02 (±2.57)	-0.34 (±3.88)	74.75 (±26.71)
	5-FU 2.4 μM	30.03 (±4.25)	8.64 (±3.32)	16.03 (±9.66)	78.59 (±23.42)
	5-FU 4.8 μM	49.22 (±3.08)	29.48 (±5.72)	30.12 (±9.22)	83.47 (±19.11)
LS513	DHA+EPA only		-28.76 (±2.11)	17.15 (±17.35)	89.91 (±7.81)
	5-FU 0.125 μM	8.93 (±2.22)	-9.42 (±2.14)	32.37 (±14.36)	91.62 (±5.90)
	5-FU 0.250 μM	25.23 (±2.32)	14.87 (±3.23)	46.34 (±11.34)	92.09 (±7.01)
	5-FU 0.500 μM	41.64 (±3.80)	37.57 (±7.29)	56.12 (±8.05)	93.22 (±4.54)

Table S7. Estimated effect of combinatory treatments of 5-FU and DHA+EPA in indicated concentrations (for DHA+EPA the OmeGo correlated concentrations were used). Values estimated by fitting the data to a linear model.

Cell line	Treatment	DHA+EPA 300	DHA+EPA 500	DHA+EPA 700
DLD-1	5-FU 1.2 μM	39.40	62.95	86.50
	5-FU 2.4 μM	48.29	67.81	87.33
	5-FU 4.8 μM	66.08	77.53	88.98
LS411N	5-FU 1.2 μM	38.66	56.50	74.34
	5-FU 2.4 μM	46.51	60.99	75.47
	5-FU 4.8 μM	62.20	69.96	77.72
HCT-8	5-FU 1.2 μM	17.83	32.22	46.61
	5-FU 2.4 μM	28.84	39.55	50.27
	5-FU 4.8 μM	50.85	54.22	57.59

**LS513**	5-FU 0.125 µM	20.90	44.68	68.45
	5-FU 0.250 µM	33.26	51.70	72.15
	5-FU 0.500 µM	57.97	68.76	79.55

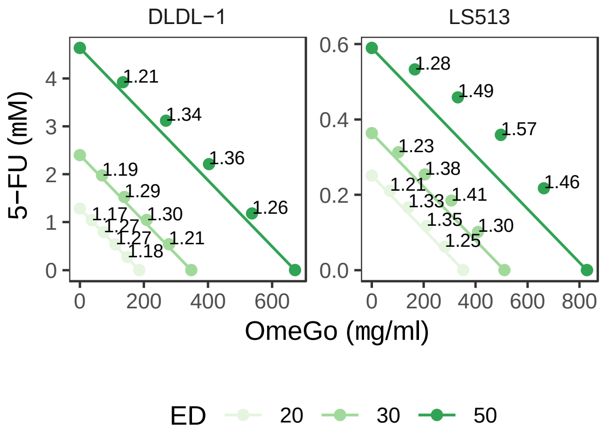

Figure S1. Combination results from 5-FU and OmeGo treatment of DLD-1 and LS513 cells. For each cell line, the corresponding drug combination linear model was used to create OmeGo and 5-FU combination values for selected effective doses. Values are computed Chou-Talalay combination indices for selected drug combination values.

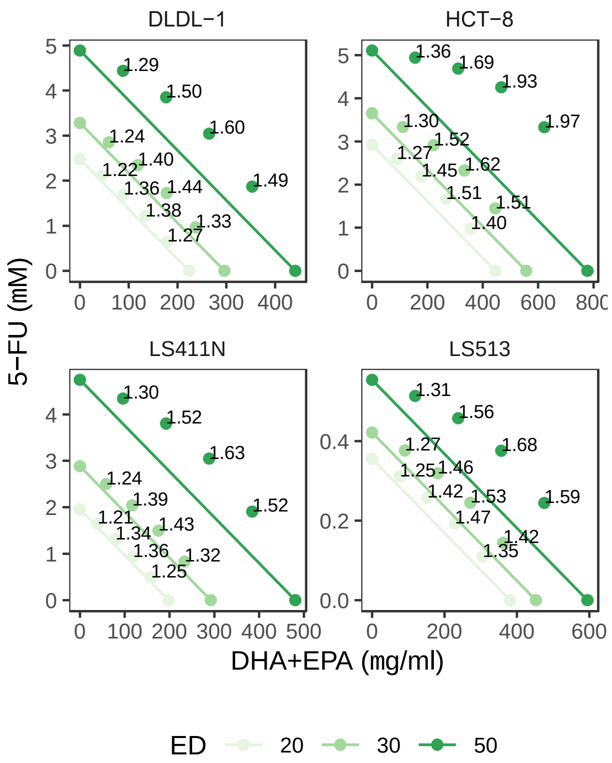

Figure S2. Combination results from 5-FU and DHA+EPA treatment of DLD-1, LS411N, HCT-8, and LS513 cells. See Figure S1 for details.
